# Supplementary material for: Human Microbiome Mixture Analysis Using Weighted Quantile Sum Regression
Source: Int J Environ Res Public Health. 2022 Dec 21;20(1):94. doi: 10.3390/ijerph20010094 (PMC9819204; doi:10.3390/ijerph20010094)
Supplement: Supplementary file 1 [file ijerph-20-00094-s001.zip › ijerph-2043630-supplementary.pdf]

Human Microbiome Mixture Analysis using Weighted Quantile Sum Regression

Shoshannah Eggers, PhD, Moira Bixby, MPH, Stefano Renzetti, PhD , Paul Curtin, PhD, Chris Gennings, PhD

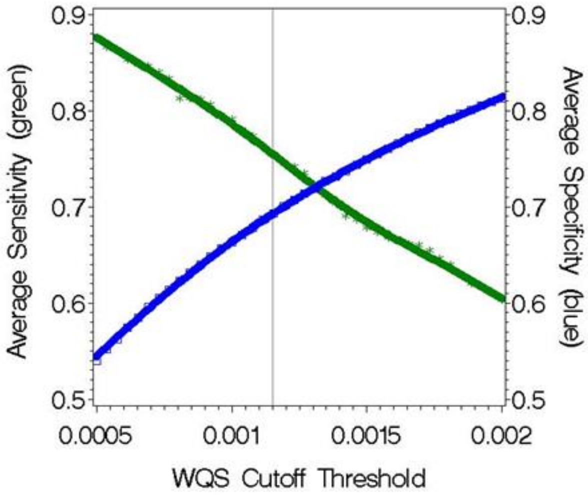

Supplemental Figure S1: **WQS Sensitivity and Specificity.** Average sensitivity and specificity based on a range of threshold cutoff values from 30 repeated holdout analyses for the Weighted Quantile Sum (WQS) operational taxonomic unit (OTU) index including 868 components.

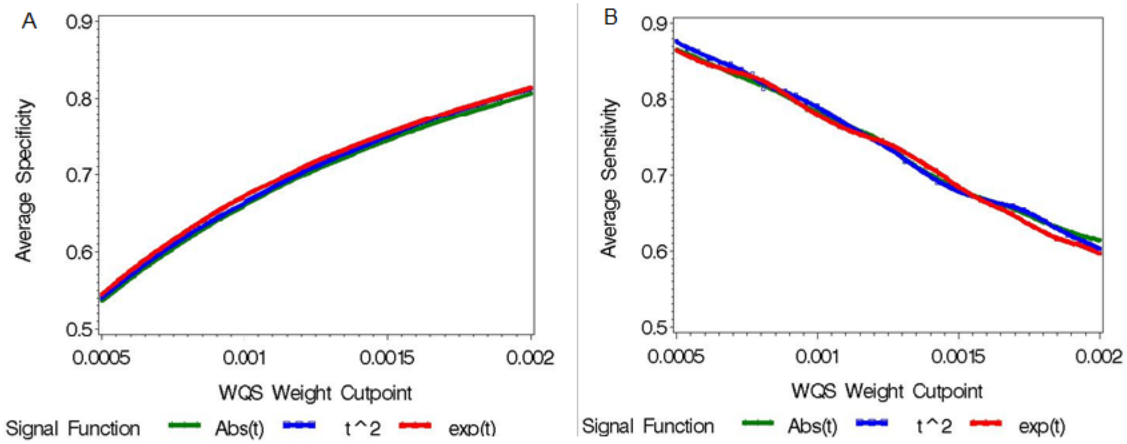

Supplemental Figure S2: LOESS plots of the average (A) specificity; and (B) sensitivity from the 30 repeated holdout datasets, across cutpoints and signal functions.
